# Assessment of Spectral-Domain Optical Coherence Tomography Findings in Three Cases of X-Linked Juvenile Retinoschisis in the Same Family

**DOI:** 10.4274/tjo.09068

**Published:** 2017-10-27

**Authors:** Sibel Doğuizi, Mehmet Ali Şekeroğlu, Salih Çolak, Mustafa Alpaslan Anayol, Pelin Yılmazbaş

**Affiliations:** 1 Ulucanlar Eye Training and Research Hospital, Ophthalmology Clinic, Ankara, Turkey

**Keywords:** Hereditary retinal dystrophy, spectral-domain optical coherence tomography, X-linked juvenile retinoschisis

## Abstract

X-linked juvenile retinoschisis (XLRS) is an X-linked hereditary retinal dystrophy characterized by splitting of the neurosensory retina. On fundus examination, the macula often has a spoke wheel appearance with foveal cystic lesions, and separation of the retinal layers is typical on spectral-domain optical coherence tomography (SD-OCT). Patients with XLRS can exhibit different clinical courses, stages, and SD-OCT findings, even among members of the same family. SD-OCT is an important imaging method that allows us to achieve more detailed information about XLRS. In this study, we report three patients in the same family who have different clinical features and SD-OCT findings.

## INTRODUCTION

X-linked juvenile retinoschisis (XLRS) is a hereditary retinal dystrophy with a prevalence ranging between 1:5,000 and 1:25,000. It is the most common form of macular degeneration among male children and adolescents, and females are carriers due to its X-linked recessive inheritance pattern.^1^ The first symptoms typically appear in school-age children 5-10 years old, and visual acuity during this period is usually between 20/200 and 20/50.^1,2^ Fundus examination often reveals a bicycle spoke wheel appearance indicating foveal schisis in the macula, and separation of the retinal layers is a typical finding on spectral-domain optical coherence tomography (SD-OCT).^1,2,3^ Peripheral retinoschisis is also seen in 50% of cases, most commonly in the inferotemporal quadrant. Electroretinography (ERG) typically shows reduced b-wave and normal a-wave, indicative of a defect in the inner retinal layers.^1,2,3^ Other clinical findings include white spots on the retina, thinning of the retinal vessels, dendritic appearance of the peripheral retina, and vascular sheathing, and complications such as vitreous hemorrhage and retinal detachment may occur.^1,2,3^

XLRS can manifest with highly variable clinical spectra, and even members of the same family may exhibit different clinical features and SD-OCT findings. SD-OCT is especially valuable because it reveals findings that cannot be distinguished in fundus examination and facilitates detection of the different phenotypic signs of the disease.^4^ In this article, we present and discuss the different clinical and SD-OCT findings of three patients from the same family.

## CASE REPORT

### Case 1

**Figure 1:**
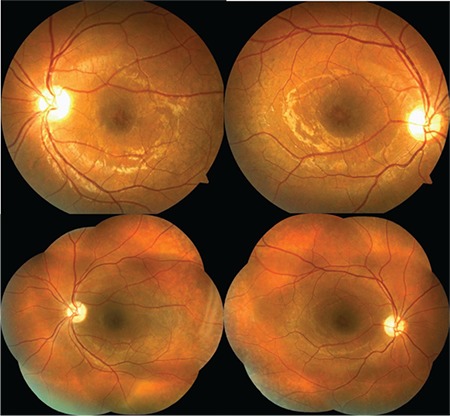
Case 1: Color fundus photographs of the left and right eyes showing bilateral spoke wheel appearance consistent with foveal schisis, pigmentary changes in the peripheral retina, and vitreous syneresis

**Figure 2:**
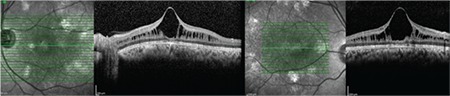
Case 1: Spectral-domain optical coherence tomography images of the left and right eyes showing marked schisis cavities in the fovea of both eyes, especially in the inner nuclear layer, with large bilateral foveal cysts

A 12-year-old male patient presented to our clinic with complaints of low vision in both eyes. His best corrected visual acuity was 20/100 in the right eye and 20/80 in the left. There was no difference in his low vision between day and night. Refraction values were +2.25 in the right and +1.25 (-1.25, 180) in the left eye. Bilateral vitreous syneresis, pigmentary changes in the peripheral retina, and a spoke wheel appearance in the macula suggesting foveal schisis were observed on dilated fundus examination (Figure 1). SD-OCT (Spectralis; Heildelberg Engineering, Heildelberg, Germany) revealed schisis cavities in the fovea of both eyes, especially prominent in the inner nuclear layer, as well as large bilateral foveal cysts (Figure 2). Central foveal thickness (CFT) was 606 μm in the right eye and 612 μm in the left eye. Deterioration in the ellipsoid zone and external limiting membrane was also observed in the foveal regions in both eyes on SD-OCT. The patient had no systemic diseases. It was indicated in his family history that his brother and uncle also had vision impairment. The patient was diagnosed with XLRS based on the findings. The patient’s other siblings and male relatives were invited for examination.

### Case 2

**Figure 3:**
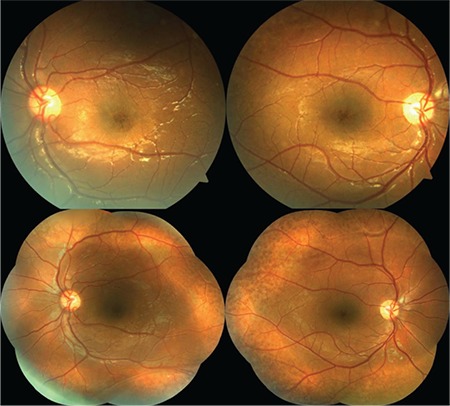
Case 2: Color fundus photographs of the left and right eyes showing bilateral macular spoke wheel appearance suggesting foveal schisis, vitreous syneresis, vitreous veils, and peripheral retinoschisis

**Figure 4:**
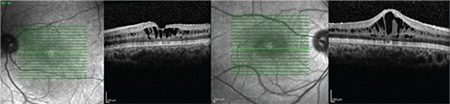
Case 2: Spectral-domain optical coherence tomography images of the left and right eyes showing cysts in the inner nuclear layer which converge form retinoschisis and continue decreasingly from the fovea to the periphery, with a few cysts in the external plexiform and outer nuclear layers

Upon examination of the siblings (1 female, 1 male) of the first patient, the 8-year-old brother was found to have bilateral low vision. His best corrected visual acuity was 20/100 in both eyes. Refraction value was +0.75 bilaterally and anterior segment examination was normal in both eyes. Bilateral vitreous syneresis, vitreous veils, peripheral retinoschisis, and macular spoke wheel appearance suggesting foveal schisis were observed on dilated fundus examination (Figure 3). SD-OCT revealed bilateral foveal cysts, especially in the inner nuclear layer, which converged to form retinoschisis. The lesions subsided from the fovea to the periphery (Figure 4). Cysts were also observed to a much lesser extent in the outer plexiform and outer nuclear layers. CFT was 324 μm in the left eye and 456 μm in the right eye. In addition, it was observed that there was irregularity in the ellipsoid zone and external limiting membrane of the foveal region, which was more pronounced in the right eye. The patient had no systemic diseases. The patient was diagnosed with XLRS based on the findings.

### Case 3

**Figure 5:**
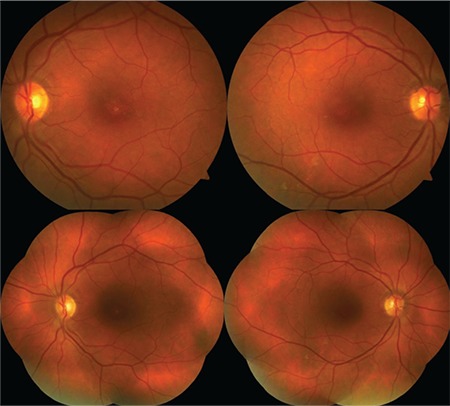
Case 3: Color fundus photographs of the left and right eyes showing bilateral macular atrophy, vitreous syneresis, and peripheral pigmentary changes

**Figure 6:**
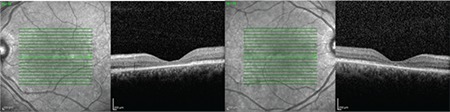
Case 3: Spectral-domain optical coherence tomography images of the left and right eyes showing marked bilateral foveal atrophy

Examination of the other male members of the family revealed bilateral low vision in the 41-year-old uncle of the first two patients. It was learned that he had suffered low vision since childhood. His best corrected visual acuity was 20/200 in both eyes. Refraction value was +0.50 bilaterally and anterior segment examination was normal in both eyes. On dilated fundus examination, there was vitreous syneresis, peripheral pigmentary changes, and a weak foveal reflex in both eyes (Figure 5). Pronounced foveal atrophy was apparent in both eyes on SD-OCT (Figure 6). CFT was 127 μm in the right eye and 125 μm in the left. It was also noted on SD-OCT that the inner retinal layers in particular could not be clearly distinguished due to severe atrophy in the foveal region. The patient had no systemic diseases. The patient was diagnosed with XLRS based on the findings.

## DISCUSSION

XLRS is an X-linked recessive retinal dystrophy characterized by splitting of the neurosensory retina into layers. The disease develops due to several different mutations of the XLRS1 gene located in the p22 region of the X chromosome.^5^ The XLRS1 gene encodes the retinoschisis protein, which is predominantly expressed by photoreceptor and Müller cells and plays a crucial role in preserving the anatomical and functional integrity of the retina. Abnormal production of this protein due to mutation disrupts the structural integrity of the retina, leading to the formation of cysts and schisis cavities, which can develop in all retinal layers.^5^

The disease is usually detected during childhood or adolescence, but can more rarely manifest in infants with nystagmus and strabismus. Fundus examination findings of microcysts with spoke wheel appearance, especially in the foveal region, is considered the main diagnostic sign. Over the course of years, this appearance may disappear, the microcysts may converge to form large foveal cysts, or foveal atrophy may develop in more advanced stages.^3,6^

Differential diagnosis of XLRS includes retinitis pigmentosa, acquired retinoschisis, Goldman-Favre syndrome, Wagner’s disease, Stickler syndrome, macular dystrophies, choroidal dystrophies, and peripheral vitreoretinal degenerations. Our cases were distinguished from these diseases based on their vision levels, clinical findings, lack of comorbid systemic diseases, presence of typical macular lesions, SD-OCT findings, and hereditary pattern.

Clinical findings and ERG are important for the diagnosis of XLRS, but SD-OCT has a critical role in showing the plane of separation in the neurosensory retina and the size and extent of the schisis cavities, especially in the early stages of the disease. Clinical features and SD-OCT findings of the disease may differ between the members of the same family, as well as between the two eyes of the same patient. In addition, the characteristics of the lesions may change over time.^7^ This variability is also apparent in our cases.

The most common SD-OCT finding in XLRS is extensive retinal splitting (schisis formation) in the inner nuclear layer. Less commonly, signs of splitting can also be seen in the outer nuclear and outer plexiform layers, and small cystoid changes have been reported in the nerve fiber-ganglion cell layer.^8,9,10^ Similarly, in our second case, SD-OCT revealed marked schisis cavities in the fovea of both eyes, especially in the inner nuclear layer, with fewer cysts in the outer plexiform and outer nuclear layers. Although schisis cavities are typically limited to the foveal pit, they may also continue on both sides and extend across the entire posterior pole; however, in our cases they were limited to the foveal region. Recent studies of this rare disease have demonstrated that the extent of schisis is greater in the inner nuclear and outer nuclear-plexiform layers, which contradicts previous histopathological studies showing that schisis occurs mostly in more superficial retinal layers such as the internal limiting membrane and nerve fibers.^8,9,10^ We believe that this discrepancy may be a result of technical limitations of the histopathological studies, and more accurate information about XLRS can be obtained using SD-OCT, which enables high-resolution *in vivo* imaging of the retina.

Our first patient exhibited large foveal cysts, which is less common in XLRS and almost always accompanied by schisis cavities in the inner nuclear layers. Large foveal cysts are thought to form when the septa between previously existing cavities rupture, and a possible association with lower visual acuity as been reported.^7,8,9^ However, the visual acuity of our first patient was similar to that of our second patient. In XLRS, phenotypic SD-OCT images characterized by intraretinal schisis formation can regress over time, giving way to atrophy.^10,11,12,13^ Likewise, in our study, SD-OCT revealed marked foveal atrophy in both eyes of the 41-year-old uncle of the other two siblings with XLRS. At this stage, the foveal atrophy detected with SD-OCT is not diagnostic on its own. However, we reached the diagnosis based on the patient’s nephews having XLRS, the patient’s history of low vision since childhood, and the detection of pigmentary changes in the peripheral retina and vitreous changes on examination.

This study presents three cases from the same family with different clinical and SD-OCT findings. XLRS may have different clinical courses and SD-OCT findings, even among individuals in the same family. SD-OCT is a crucial, unparalleled imaging method that allows us to understand the different structural changes caused by the disease in the macula, and elucidate the levels and stages of these defects.
